# Effect of rhegmatogenous retinal detachment on preoperative and postoperative retinal sensitivities

**DOI:** 10.1038/s41598-020-78693-5

**Published:** 2020-12-09

**Authors:** Hiroshi Noda, Shuhei Kimura, Mio Morizane Hosokawa, Yusuke Shiode, Shinichiro Doi, Kosuke Takahashi, Ryo Matoba, Yuki Kanzaki, Atsushi Fujiwara, Yuki Morizane

**Affiliations:** Department of Ophthalmology, Okayama University Graduate School of Medicine, Dentistry and Pharmaceutical Sciences, 2-5-1 Shikata-cho Kita-ku, Okayama, Okayama 700-8558 Japan

**Keywords:** Medical research, Diseases

## Abstract

This retrospective study investigated foveal and perifoveal retinal sensitivities using microperimetry before and after surgery for rhegmatogenous retinal detachment (RRD). Consecutive patients with RRD who underwent vitrectomy or scleral buckling were included. Comprehensive ophthalmological examinations, including microperimetry and swept-source optical coherence tomography, were performed before and 6 months after surgery. Pre- and postoperative retinal sensitivities at the fovea and 4 perifoveal measurement points farthest from the fixation point, both vertically and horizontally (superior, inferior, nasal, and temporal) were examined. A total of 34 foveal and 136 perifoveal measurement points in 34 eyes of 34 patients were evaluated. The postoperative retinal sensitivity was significantly higher than the preoperative value at foveal and perifoveal points with (*P* < 0.001 for both) and without (fovea: *P* = 0.005, perifovea: *P* < 0.001) RRD. The postoperative retinal sensitivity was significantly lower at foveal (*P* < 0.01) and perifoveal (*P* < 0.001) points with preoperative RRD than at points without preoperative RRD; furthermore, it was significantly better at points with ellipsoid zone (Ez) continuity than at points with Ez discontinuity (fovea: *P* < 0.01, perifovea: *P* < 0.001). RRD deteriorates retinal sensitivity, regardless of its presence or absence at the measurement point before surgery. Postoperative Ez continuity is important for good postoperative retinal sensitivity.

## Introduction

Previous evaluations of visual function in cases of rhegmatogenous retinal detachment (RRD) were primarily based on visual acuity^1–3^. However, because visual function at the fovea as well as the area outside the fovea is disturbed in cases of RRD, evaluation of visual acuity is not sufficient to describe visual function in these cases. It has been reported that retinal function at the perifovea (the area forming the central 10 degrees of the retina) is particularly important for maintaining the quality of vision. In fact, diseases that disturb the perifoveal retinal sensitivity, such as glaucoma and retinitis pigmentosa, can severely affect daily activities such as walking, driving, and reading^4,5^.

In cases of RRD, the perifoveal retinal sensitivity has been analyzed using a microperimeter^6,7^. This device facilitates measurement of the perifoveal retinal sensitivity over a wide range of stimulus luminances (0–36 dB). Furthermore, it enables analysis of the relationship between the retinal lesion location and retinal sensitivity by projecting a fundus image on the retinal sensitivity map, in addition to accurate evaluation of changes in retinal sensitivity over time with an eye tracking system and follow-up function^8,9^. However, to our knowledge, no study has investigated the effects of RRD on the preoperative perifoveal retinal sensitivity and the relationship between the pre- and postoperative perifoveal retinal sensitivities. Furthermore, factors related to changes in the perifoveal retinal sensitivity in eyes with RRD are unknown.

Therefore, the aim of the present study was to investigate foveal and perifoveal retinal sensitivities using a microperimeter before and after surgery for RRD. Furthermore, we focused on the outer retinal microstructure after surgery and investigated its relationship with the postoperative retinal sensitivity.

## Results

Of the 52 reviewed eyes, 18 eyes were excluded [17 eyes whose retinal detachment did not spread to the perifovea and 1 eye with concomitant epiretinal membrane (ERM)]. Thus, 34 eyes of 34 patients with RRD involving the perifovea were included (see Supplementary Table S1). In all patients, OCT imaging was performed on the day of presentation and surgery was performed on the day of, or the day after presentation. Twenty-five (74%) and 9 (26%) eyes underwent pars plana vitrectomy (PPV) and scleral buckling (SB), respectively, and the retina was successfully attached after the initial surgery in all eyes. Among the 25 eyes that underwent PPV, 17 (50%) received simultaneous cataract surgery. None of the eyes that underwent SB received cataract surgery. There were no significant differences in age, sex, affected side, and surgical procedure between the fovea-on and fovea-off groups (Table 1). The preoperative logarithm of the minimal angle of resolution (logMAR) best-corrected visual acuity (BCVA) for eyes with fovea-on RRD was significantly better than that for eyes with fovea-off RRD (− 0.00 ± 0.09 vs 0.83 ± 0.54; *P* < 0.001). This result was also observed for the postoperative BCVA (fovea-on: − 0.07 ± 0.05 vs fovea-off: 0.14 ± 0.27; *P* = 0.017).

**Table 1:** Characteristics of patients with rhegmatogenous retinal detachment.

Preoperative RD at the macula	No	Yes	*P*-Value
Number of eyes	11	23	
Age (years)	60.0 ± 6.1	52.5 ± 18.9	0.21
Sex (male/female)	8/3	16/7	0.85
OD/OS	5/6	12/11	0.71
Surgery (SB/PPV/PPV + PEA + IOL)	1/2/8	8/6/9	0.13
Preoperative BCVA	− 0.00 ± 0.09	0.83 ± 0.53	< 0.001
Postoperative BCVA	− 0.07 ± 0.05	0.14 ± 0.27	0.017

*BCVA* best corrected visual acuity, *IOL* intraocular lens, *OD* oculus dexter, *OS* oculus sinister, *PEA* phacoemulsification and aspiration, *PPV* pars plana vitrectomy, *RD* retinal detachment, *SB* scleral buckling.

**Figure 1:**
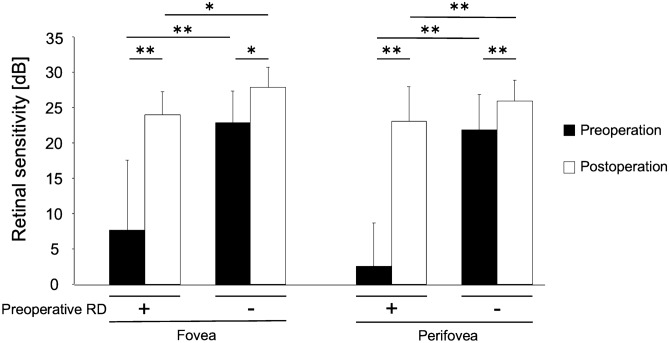
Changes in retinal sensitivity at the measurement points with or without preoperative retinal detachment in eyes with rhegmatogenous retinal detachment. At both the fovea and perifovea, the postoperative retinal sensitivity is significantly better than the preoperative retinal sensitivity, regardless of the presence of preoperative retinal detachment at the measurement points. The postoperative retinal sensitivity at the measurement points with preoperative retinal detachment is significantly worse than that at measurement points without preoperative retinal detachment. *RD* retinal detachment; *P < 0.01; **P < 0.001.

In total, 34 foveal and 136 perifoveal measurement points in the 34 eyes were evaluated. Pre- and postoperative retinal sensitivity values at measurement points with preoperative retinal detachment (23 foveal points and 77 perifovea points, including 16 superior points, 25 inferior points, 12 nasal points, and 24 temporal points) were compared. The postoperative retinal sensitivity was significantly better than the preoperative retinal sensitivity at the 23 foveal measurement points with preoperative RRD (24.0 ± 3.3 dB vs 7.7 ± 9.9 dB; *P* < 0.001; Fig. 1). The same result was observed for the 77 perifoveal measurement points with preoperative RRD (postoperative: 23.1 ± 4.9 dB vs preoperative: 2.6 ± 6.0 dB; *P* < 0.001; Fig. 1).

Next, pre- and postoperative retinal sensitivity values at measurement points without preoperative retinal detachment (11 foveal points and 59 perifoveal points, including 18 superior points, 9 inferior points, 22 nasal points, and 10 temporal points) were compared. The postoperative retinal sensitivity was significantly better than the preoperative retinal sensitivity at the 11 foveal (27.9 ± 2.9 dB vs 22.9 ± 4.5 dB; *P* = 0.005; Fig. 1) and 59 perifoveal (26.1 ± 3.0 dB vs 21.9 ± 4.9 dB; *P* < 0.001) measurement points without preoperative RRD.

To exclude the effect of cataract surgery on retinal sensitivity changes, the patients selected did not receive simultaneous cataract surgery and the pre- and postoperative retinal sensitivity values at measurement points without preoperative retinal detachment were compared. Seventeen patients did not receive cataract surgery, and there were 3 foveal and 22 perifoveal measurement points without preoperative retinal detachment in these patients. Because the number of foveal measurement points was small, we focused on the perifoveal measurement points and performed the comparison. The postoperative perifoveal retinal sensitivity was significantly better than the preoperative sensitivity; this indicated that the effect of cataract surgery on retinal sensitivity changes was negligible (25.2 ± 2.5 dB vs 20.2 ± 6.9 dB; *P* = 0.001; see Supplementary Fig. S1).

The influence of preoperative retinal detachment on the postoperative retinal sensitivity was analyzed, which was significantly lower at points with retinal detachment than at points without retinal detachment at the fovea (24.0 ± 3.3 dB vs 27.9 ± 2.9 dB; *P* < 0.01; Fig. 1) and perifovea (23.1 ± 4.9 dB vs 26.1 ± 3.0 dB; *P* < 0.001; Fig. 1).

**Figure 2:**
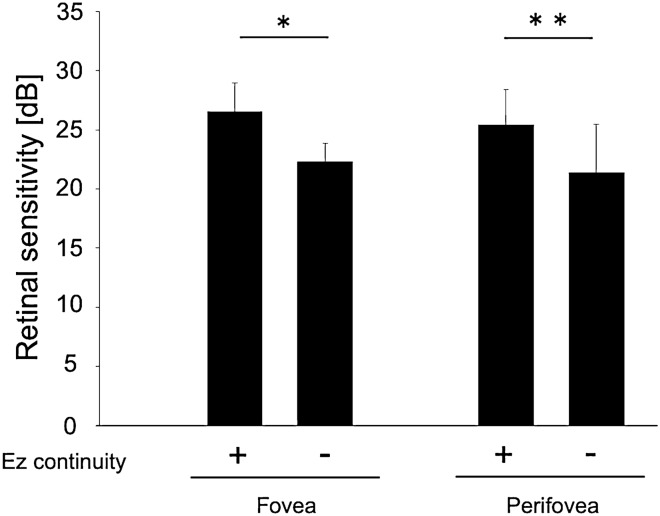
Relationship between the postoperative retinal structure and retinal sensitivity in eyes with rhegmatogenous retinal detachment. At both the fovea and perifovea, the postoperative retinal sensitivity is significantly better in the group with ellipsoid zone (Ez) continuity than in the group without Ez continuity. Eyes with postoperative subretinal fluid are excluded from this analysis. *P < 0.01; **P < 0.001.

To determine the influence of the postoperative outer retinal microstructure, particularly the continuity of the ellipsoid zone (Ez), on the postoperative foveal and perifoveal retinal sensitivity, 30 foveal and 128 perifoveal measurement points after excluding points where subretinal fluid (SRF) was observed on swept-source optical coherence tomography (SS-OCT) images after surgery (4 foveal and 8 perifoveal points) were analyzed. At the fovea, the postoperative retinal sensitivity was significantly better at measurement points with Ez continuity (Ez+: 27 measurement points, 26.6 ± 2.4 dB) than at points with Ez discontinuity (Ez−: 3 measurement points, 22.3 ± 1.5 dB; P < 0.01; Fig. 2). The same result was observed for the perifoveal points (Ez+: 114 measurement points, 25.4 ± 2.9 dB vs Ez−: 14 measurement points, 21.4 ± 4.1 dB; P < 0.001; Fig. 2).

**Figure 3:**
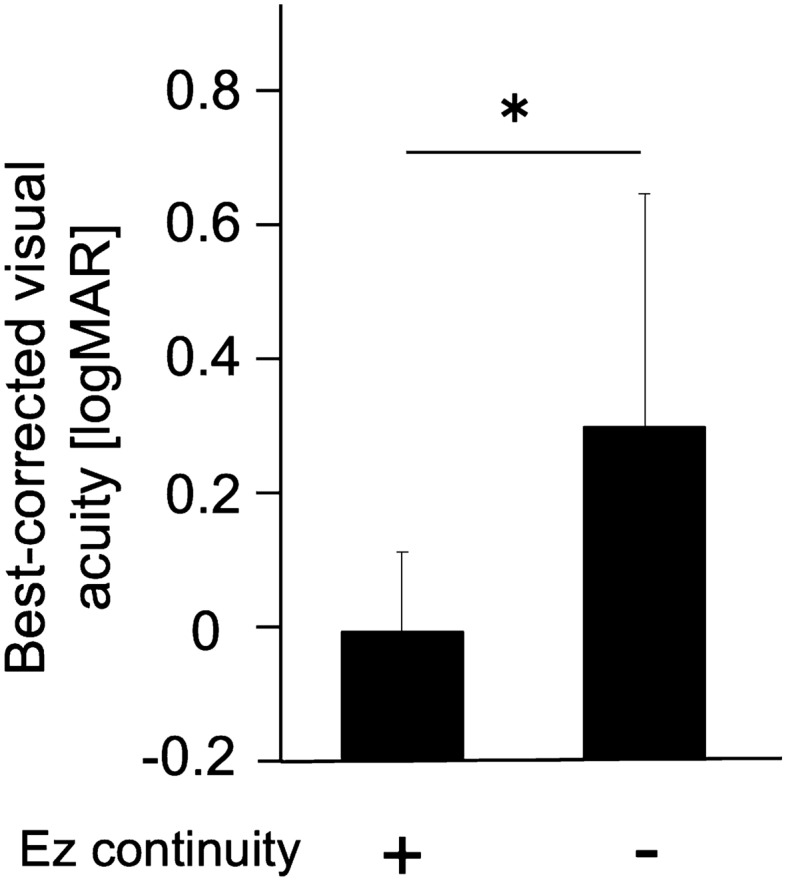
Relationship between the postoperative retinal structure and postoperative best-corrected visual acuity (BCVA) in eyes with rhegmatogenous retinal detachment. The postoperative BCVA is significantly better in the group with ellipsoid zone (Ez) continuity than in the group without Ez continuity. Eyes with postoperative subretinal fluid are excluded from this analysis. logMAR: logarithm of the minimum angle of resolution; *P < 0.01.

There was a negative correlation between the postoperative foveal retinal sensitivity and the postoperative logMAR BCVA (r = 0.57, *P* < 0.01; see Supplementary Fig. S2). After exclusion of 4 eyes with postoperative SRF at the fovea, the postoperative BCVA for eyes with Ez+ was significantly better than that for eyes with Ez− (− 0.01 ± 0.12 vs 0.30 ± 0.35; *P* < 0.01; Fig. 3).

To determine the influence of surgical procedure, 34 eyes (vitrectomy group 25 eyes, SB group 9 eyes) were analyzed. Excluding the measurement points where SRF remained postoperatively, 30 foveal measurement points (vitrectomy group 24 points, SB group 6 points) and 128 perifoveal measurement points (vitrectomy group 98 points, SB group 30 points) were analyzed. There was no significant difference in postoperative retinal sensitivity between the vitrectomy surgery and SB at the foveal measurement points with preoperative retinal detachment (*P* = 0.20). At the perifoveal measurement points, there was no significant difference in postoperative retinal sensitivity between the vitrectomy surgery and SB, whether preoperative retina was on or off (*P* = 0.43, 0.49, respectively). Because only 1 eye underwent SB without preoperative foveal detachment, the statistical analysis of postoperative retinal sensitivity between the vitrectomy surgery and SB at foveal measurement point could not performed.

**Figure 4:**
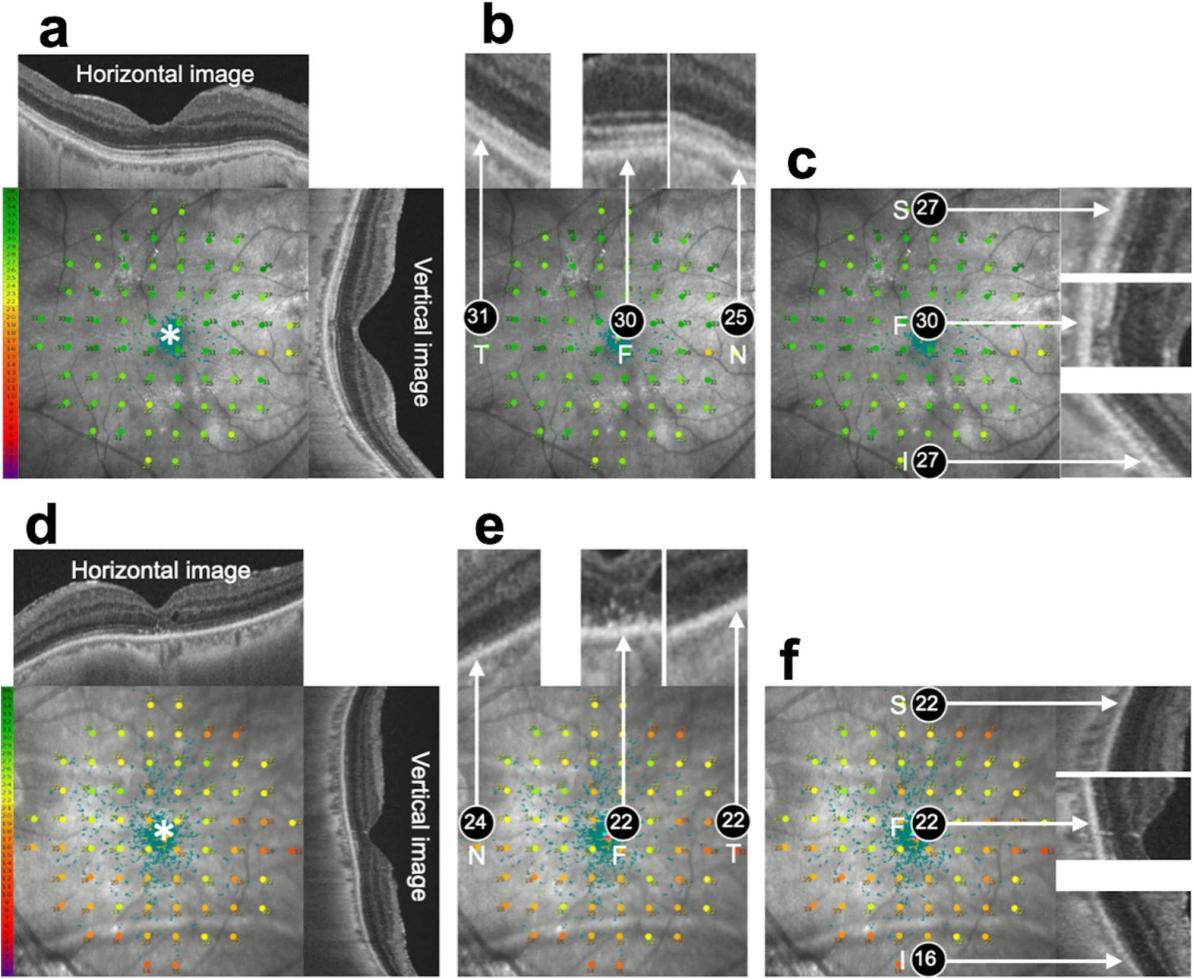
Representative cases with or without postoperative ellipsoid zone (Ez) continuity after surgery for rhegmatogenous retinal detachment (RRD). (**a**–**c**) The patient is a 57-year-old man with fovea-on RRD. The retina is detached at the superior measurement points, but not at the other 4 measurement points (foveal, temporal, nasal, and inferior). His preoperative best-corrected visual acuity (BCVA) is − 0.08. The preoperative foveal, temporal, nasal, superior, and inferior retinal sensitivity values for this case are 27, 25, 23, 0, and 21 dB, respectively. Six months after the surgery, the postoperative BCVA of this patient is maintained at − 0.08. Postoperative fundus images obtained using Macular Integrity Assessment system (MAIA) and swept-source optical coherence tomography (SS-OCT) are superimposed (**a**). The postoperative foveal, temporal, nasal, superior, and inferior retinal sensitivity values are 30, 31, 25, 27, and 27 dB, respectively (**b**,**c**). B-scan images corresponding to the 5 measurement points show that Ez is continuous at all measurement points (**b**,**c**). (**d**–**f**) The patient is a 65-year-old woman with RRD. The retina is detached at all measurement points. Her preoperative BCVA is 1.70. The preoperative retinal sensitivity values at all measurement points are 0. Six months after the surgery, the postoperative BCVA is 0.70. Postoperative fundus images obtained using MAIA and SS-OCT are superimposed (**d**). The postoperative foveal, nasal, temporal, superior, and inferior retinal sensitivity values are 22, 24, 22, 22, and 16 dB, respectively (**e**,**f**). B-scan images corresponding to the 5 measurement points show that Ez is continuous at the nasal measurement point whereas Ez is discontinuous at the other measurement points (fovea, temporal, superior, inferior, **e** and **f**). Asterisk (*): fixation point; *F* fovea, *S* superior, *I* inferior, *T* temporal, *N* nasal. Numbers in the circles represent the retinal sensitivity (dB) at each measurement point.

Figure 4a–c shows a representative case with postoperative Ez+, while Fig. 4d–f shows a representative case with postoperative Ez−.

## Discussion

Although past studies have evaluated the postoperative foveal retinal sensitivity in cases of RRD^7,10–15^, to the best of our knowledge, the present study is the first to investigate the pre- and postoperative retinal sensitivities in cases of RRD. We also investigated the changes in retinal sensitivity at both the fovea and perifovea along with their associations with the preoperative presence of retinal detachment and the postoperative outer retinal structure. The main findings were as follows. First, the postoperative retinal sensitivity at measurement points with preoperative RRD was significantly lower than that at points without preoperative RRD. Second, when retinal detachment occurred, the preoperative retinal sensitivity was significantly lower than the postoperative retinal sensitivity, even at measurement points without RRD before surgery. Third, postoperative Ez+ was found to be important for good postoperative retinal sensitivity.

With regard to the relationship between the postoperative continuity of Ez and the postoperative retinal sensitivity, our result agreed well with those of past studies reporting the presence of correlations between decreased postoperative retinal sensitivity and disruption of the outer retinal structure at the fovea in RRD^6,7^. These results suggest that RRD must be treated before the outer retinal structure is damaged in order to achieve good postoperative retinal sensitivity.

This study found that retinal detachment caused the preoperative retinal sensitivity to be significantly lower than the postoperative retinal sensitivity, even when preoperative RRD was not present at the measurement point. This result was in agreement with those of past studies comparing retinal function between eyes with fovea-on RRD and fellow eyes^16,17^. For example, Okamoto et al.^16^ showed that the pre- and postoperative contrast sensitivities were significantly lower in eyes with macula-on RRD than in the fellow eyes. Furthermore, Akiyama et al.^17^ performed focal macular electroretinography (ERG) and found that the amplitudes of a- and b-waves and oscillatory potentials were significantly smaller for eyes with macula-on RRD than for fellow eyes. Although the mechanism underlying these results is unknown, it has been discussed that retinal ischemia at the detached areas in eyes with macula-on RRD reduces the macular blood flow via the upregulation of endothelin-1, leading to reduced ERG responses^16,17^, Eshita et al.^18^ measured the macular blood flow in 28 patients with macula-n RRD using scanning laser doppler flowmetry and found that the mean blood flow ratio in the affected eye was lower than that in the fellow eye both before and after the surgery.

This study has several important limitations. First, the study design was retrospective. Second, the sample was small size and included high myopia eyes. Third, the follow-up period was relatively short for assessing the long-term retinal sensitivity after surgery. Fourth, although 17 eyes (50%) received simultaneous cataract surgery, the effect of cataract surgery on retinal sensitivity was not considered. However, a subgroup analysis was conducted for the eyes without cataract surgery and revealed that the postoperative retinal sensitivity at the perifovea was significantly better than the preoperative retinal sensitivity (see Supplementary Fig. S1). Fifth, we did not consider the duration of detachment and the amount of SRF because we could not obtain objective and quantitative data for them.

In conclusion, the results of this study suggest that RRD deteriorates retinal sensitivity, regardless of its presence or absence at the measurement point before surgery. In addition, postoperative Ez continuity is important for good postoperative retinal sensitivity.

## Methods

We retrospectively reviewed the records for 52 consecutive eyes of 52 patients with RRD who underwent surgical treatment at Okayama University Hospital between January 1st and June 30th, 2018 and were followed up for at least 6 months. Among the 52 eyes, eyes whose retinal detachment did not extend to perifovea was excluded. The eyes with other ocular diseases, such as ERM, macular hole, age-related macular degeneration, and retinal vein occlusion were also excluded.

This study was approved by the Ethics Committee of Okayama University Hospital, Okayama, Japan, and the study adhered to the tenets of the Declaration of Helsinki. Each patient was informed about the risks and benefits of the surgery before they provided written informed consent.

### Ophthalmological examinations

All patients underwent comprehensive ophthalmological examinations before and 6 months after surgery. These examinations included measurements of the BCVA with refraction using the 5-m Landolt C acuity chart, indirect and contact lens slit-lamp biomicroscopy, microperimetry, and SS-OCT. The extent of retinal detachment was determined by combining the results of ophthalmoscopy, color photographs, and OCT imaging.

### Microperimetry

**Figure 5:**
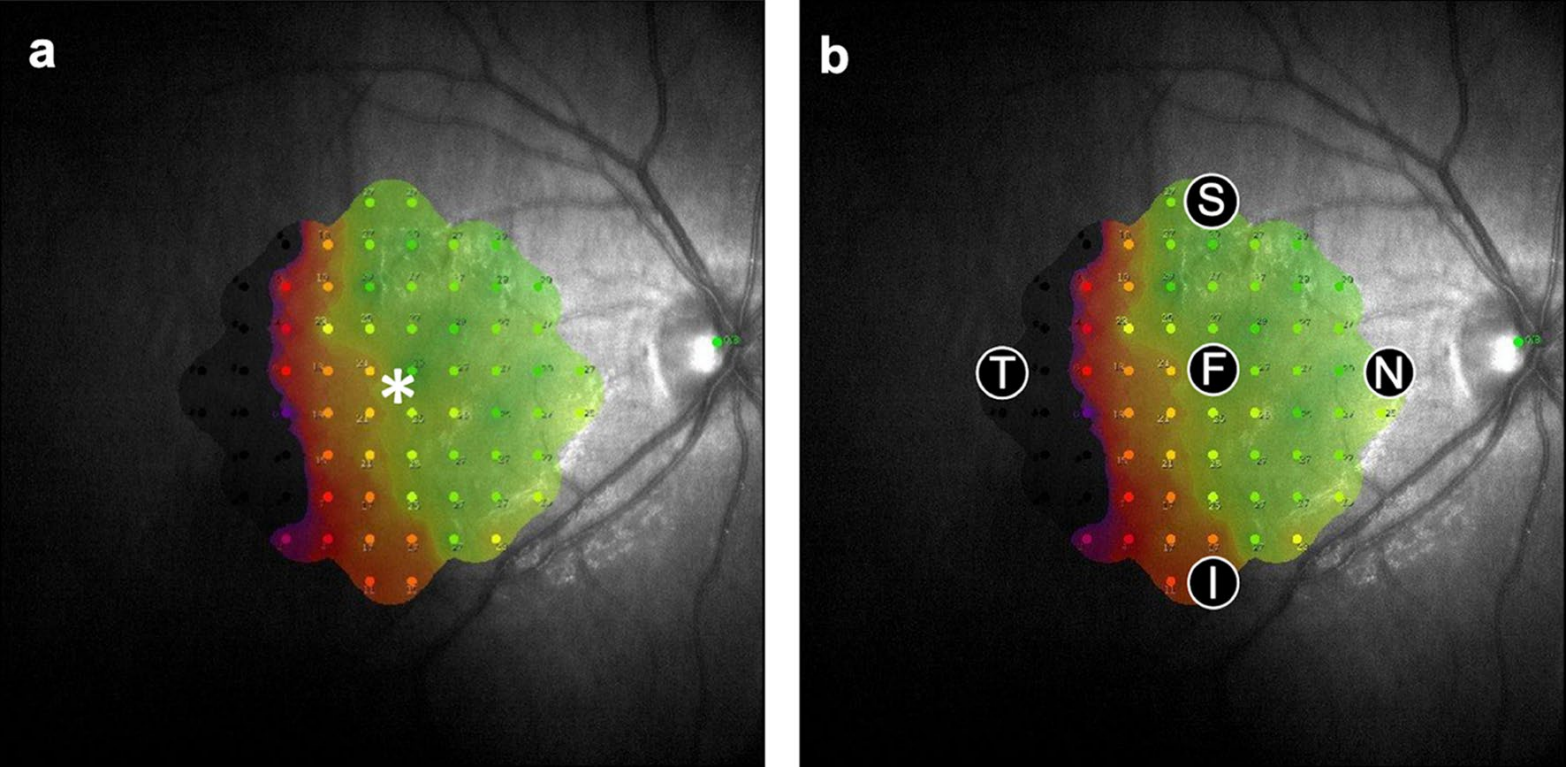
Definition of foveal and perifoveal points for the measurement of retinal sensitivity. The retinal sensitivity is measured using the Macular Integrity Assessment system. The foveal measurement point [F in (**b**)] is defined as the measurement point closest to the fixation point [asterisk in (**a**)]. The perifoveal measurement points are defined as the measurement points farthest from the fixation point, both horizontally and vertically [*S* superior, *I* inferior, *T* temporal; *N* nasal; in (**b**)].

The retinal sensitivities before and 6 months after the surgery were measured using the Macular Integrity Assessment system (MAIA, CenterVue, Padova, Italy). Using the eye tracking system and follow-up function of MAIA, measurements were performed with the pupil dilated in a dim room. The measurement configurations were as follows: a 68-stimuli grid covering the central 10 degrees of the retina, a stimulus size of 0.43 degrees (equivalent to Goldmann III), a stimulus dynamic range of 0–36 dB (0.25–1000 asb), stimulus presentation at 0.2 s, a 4-to-2 threshold strategy, a background luminance set at 4 asb, and a fixation target consisting of a red circle with a 0.5° diameter. We defined foveal retinal sensitivity as the retinal sensitivity at the measurement point nearest to the fixation point (Fig. 5). Perifoveal retinal sensitivities were defined as values obtained at measurement points farthest from the fixation point, both vertically and horizontally (superior, inferior, nasal, and temporal; Fig. 5). Only eyes with RRD spreading to the perifovea were included.

### Swept-source optical coherence tomography

The retinal structures were visualized, both horizontally and vertically, in the sitting position using SS-OCT (Triton, TOPCON Corporation, Tokyo, Japan) before and 6 months after the surgery. All SS-OCT images were analyzed by 3 masked retinal specialists (H.N., S.K., and Y.S.).

### Analysis of retinal structures at points of retinal sensitivity measurement.

**Figure 6:**
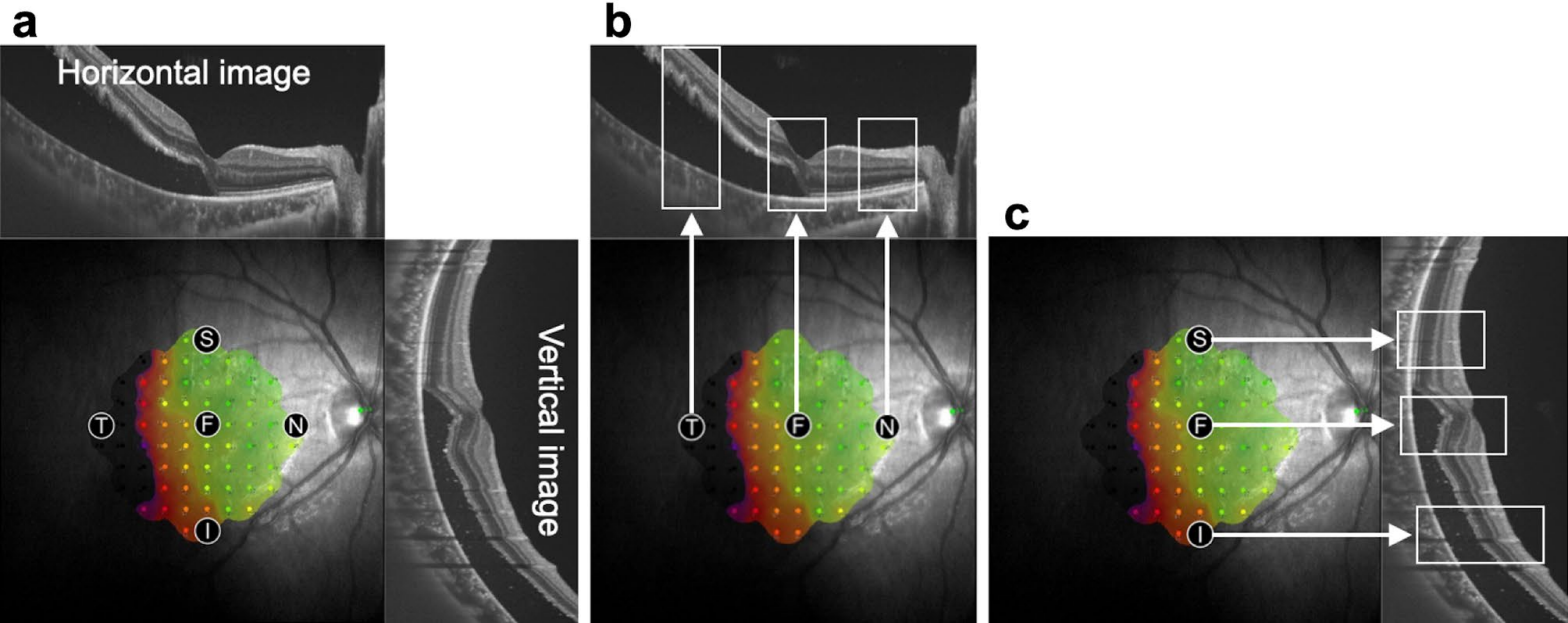
Analysis of retinal structures at the measurement points of retinal sensitivity. Fundus images obtained via Macular Integrity Assessment system and swept-source optical coherence tomography are superimposed (**a**). B-scan images corresponding to each measurement point [squares in (**b**) and (**c**)] are examined to evaluate the presence or absence of retinal detachment and the continuity of the ellipsoid zone. *F* fovea, *S* superior, *I* inferior, *T* temporal, *N* nasal.

To identify the retinal structures at the foveal and perifoveal measurement points, fundus images obtained via MAIA and SS-OCT were superimposed (Fig. 6a). Then, B-scan images corresponding to each measurement point were examined to evaluate the presence or absence of retinal detachment and the continuity of Ez (Fig. 6b,c).

### Surgical procedure

On the basis of the surgeon’s judgment, each patient underwent PPV or SB. PPV involved a 25-gauge transconjunctival microincision and was performed using the Constellation Vision System (Alcon Laboratories, Inc., Fort Worth, TX, USA). The vitreous traction around the retinal breaks was released and the SRF was drained from a posterior drainage retinotomy. Then, fluid-20% sulfur hexafluoride gas exchange and endolaser photocoagulation of the retinal breaks and intentional retinal hole were performed. The epiretinal membrane or internal limiting membrane were not peeled. Patients over 50 years old also underwent simultaneous cataract extraction with posterior chamber intraocular lens implantation^19^.When SB was performed, chorioretinal adhesions were created with cryopexy around the retinal breaks. A silicone explant was used to close the peripheral retinal breaks, and external drainage of SRF was performed when necessary. No patients were administered intravitreal injections of gas.

### Main outcome measures

The main outcome measures were the pre- and postoperative foveal and perifoveal retinal sensitivities, as well as BCVA and the microstructure of the retina at 6 months after surgery.

### Statistical analysis

All statistical methods are specified in the relevant sections of the results. BCVA values were recorded as decimal values and converted to logMAR units for statistical analysis. All visual acuity values are presented as logMAR units with Snellen equivalents in parentheses. All statistical analyses were performed using SPSS ver. 22.0 (IBM, Armonk, NY, USA). For evaluation of the surgical outcomes, postoperative BCVA and retinal sensitivity values were compared using a paired *t* test, unpaired *t* test, or one-way analysis of variance with Bonferroni correction. Correlations between the postoperative foveal sensitivity and postoperative BCVAs were performed using Spearman’s correlation analysis. Categorical data were analyzed using Fisher’s exact test. A *P*-value of < 0.05 was considered statistically significant. Data are presented as mean ± standard deviation.

## Supplementary Information

Supplementary Information.

## Data Availability

All data generated or analysed during this study are included in this published article and its Supplementary Information.
