# Supplementary material for: Effect of rhegmatogenous retinal detachment on preoperative and postoperative retinal sensitivities
Source: Sci Rep. 2020 Dec 9;10:21497. doi: 10.1038/s41598-020-78693-5 (PMC7725826; doi:10.1038/s41598-020-78693-5)
Supplement: Supplementary file 1 — Supplementary Information. [file 41598_2020_78693_MOESM1_ESM.pdf]

Effect of rhegmatogenous retinal detachment on pre- and postoperative retinal sensitivities

Hiroshi Noda, MD; Shuhei Kimura, MD, PhD; Mio Morizane Hosokawa, MD, PhD; Yusuke Shiode, MD, PhD; Shinichiro Doi, MD, PhD; Kosuke Takahashi, MD, PhD; Ryo Matoba, MD, PhD; Yuki Kanzaki, MD; Atsushi Fujiwara, CO, PhD; Yuki Morizane, MD, PhD

Institutional Affiliation:

Department of Ophthalmology, Okayama University Graduate School of Medicine, Dentistry and Pharmaceutical Sciences, 2-5-1 Shikata-cho Kita-ku, Okayama City, Okayama 700-8558, Japan

Supp.

Figure S1

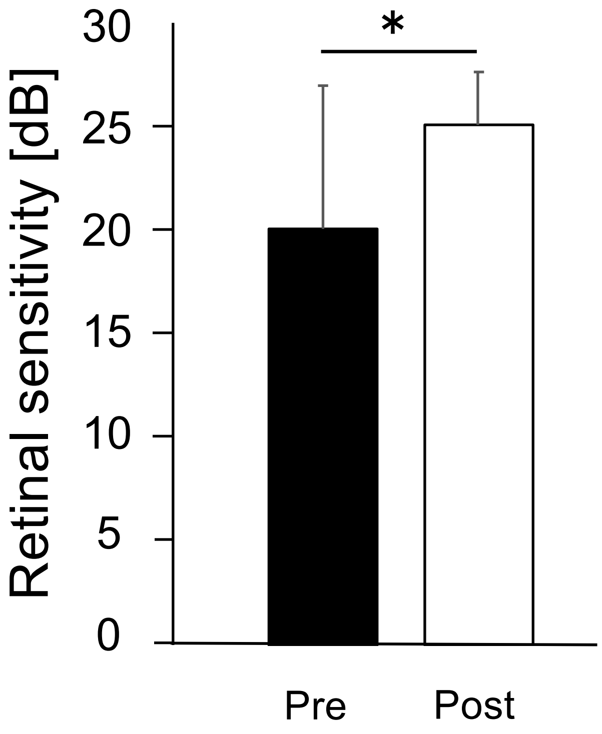

Supp.

Figure S2

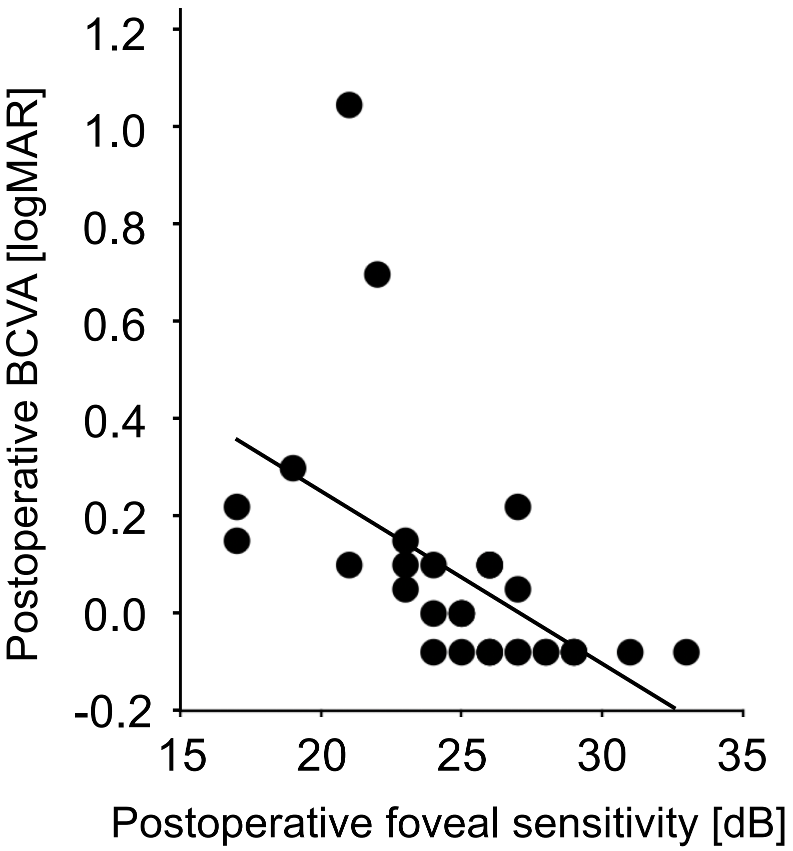

Supp. Table S1

Patient No.	Age	Sex	OD/OS	Extent of detached retina (quadrants)	AL (mm)	Operation	External drainage of SRF	Pre retina on/off					Pre retinal sensitivity (dB)					Post retinal sensitivity (dB)					Post Ez continuity					Pre logMAR	Post logMAR
								Fovea	S	I	N	T	Fovea	S	I	N	T	Fovea	S	I	N	T	Fovea	S	I	N	T		
1	65	F	OS	2	23.58	PPV+PI	NA	off	off	off	off	off	0	0	0	0	0	22	22	16	24	22	-	-	-	+	-	1.40	0.70
2	71	M	OS	2	21.96	PPV	NA	off	off	off	off	off	0	0	0	0	0	21	25	23	25	23	-	+	+	+	+	2.00	0.10
3	32	M	OS	3	25.17	SB	+	off	off	off	off	off	0	0	0	0	0	25	26	24	26	28	+	+	+	+	+	1.00	0.00
4	62	F	OS	2	23.65	PPV+PI	NA	off	off	off	off	off	0	0	0	0	0	23	25	23	27	23	+	+	+	+	+	1.70	0.10
5	69	F	OS	2	25.41	PPV	NA	off	off	off	off	off	0	0	0	0	0	26	28	26	22	24	+	+	+	+	+	0.82	0.10
6	72	M	OD	3	23.71	PPV+PI	NA	off	off	off	off	off	0	0	0	0	0	28	27	23	24	28	+	+	+	+	+	1.22	-0.08
7	15	F	OS	1	NA		-	off	off	off	off	off	0	0	0	0	0	19	21	25	27	25	SRF	SRF	-	-	-	1.40	0.30
8	64	M	OS	2	28.89	PPV	NA	off	off	off	off	off	0	0	0	14	0	23	27	12	22	25	+	+	+	+	+	0.22	0.15
9	55	F	OD	2	24.11	PPV+PI	NA	off	off	off	off	off	0	0	0	16	0	25	22	23	27	26	+	SRF	+	+	+	0.52	0.00
10	66	M	OS	2	25.55	PPV+PI	NA	off	off	on	on	off	0	0	16	17	0	25	24	28	28	24	+	+	+	+	+	0.82	-0.08
11	59	M	OD	1	NA	PPV	NA	off	off	on	on	off	0	0	18	4	0	26	25	23	23	23	+	+	+	+	+	0.52	0.10
12	55	M	OD	2	25.66	PPV+PI	NA	off	off	on	on	off	8	0	22	23	0	26	28	27	26	28	+	+	+	+	+	0.52	0.10
13	62	M	OD	2	24.41	PPV	NA	off	on	off	on	off	0	17	0	4	0	24	25	24	26	27	+	+	+	+	+	1.00	-0.08
14	63	M	OS	2	25.66	PPV+PI	NA	off	on	off	on	off	12	15	0	23	0	27	29	25	26	23	+	+	+	+	+	0.52	0.05
15	37	M	OD	1	27.00	SB	+	off	on	off	on	off	4	26	0	14	0	24	25	11	20	17	-	+	SRF	-	-	0.82	0.10
16	60	F	OS	2	22.52	PPV+PI	NA	off	on	off	on	off	24	26	15	27	17	27	25	21	23	25	+	+	+	+	+	0.40	0.22
17	74	M	OD	3	NA	PPV	NA	off	on	off	off	off	26	24	0	18	6	24	24	25	22	27	+	+	+	+	+	0.70	0.00
18	16	M	OD	2	26.38	SB	+	off	on	off	on	off	23	27	13	26	0	29	27	25	27	25	+	+	+	+	+	0.00	-0.08
19	31	F	OD	1	NA	SB	-	off	on	off	on	off	18	23	0	23	0	26	20	19	26	22	+	+	-	+	SRF	0.15	-0.08
20	33	M	OD	2	24.71	SB	-	off	on	off	on	off	25	27	0	24	0	27	30	9	26	13	+	+	SRF	+	-	0.00	-0.08
21	21	M	OS	2	25.36	SB	-	off	off	off	off	off	9	11	0	9	13	21	25	23	27	25	SRF	+	SRF	+	-	1.52	1.05
22	56	M	OD	2	26.04	SB	-	off	on	off	on	off	9	23	0	20	0	17	25	23	25	25	SRF	+	-	+	-	0.70	0.15
23	70	M	OS	2	23.56	PPV+PI	NA	off	on	off	on	off	20	23	0	23	0	17	18	17	22	21	SRF	+	SRF	+	+	1.00	0.22
24	62	F	OS	1	23.91	PPV+PI	NA	on	on	on	on	on	27	25	24	25	25	29	29	27	26	27	+	+	+	+	+	0.00	-0.08
25	56	F	OD	1	23.57	PPV+PI	NA	on	off	on	on	on	23	0	23	25	24	27	23	25	25	29	+	+	+	+	+	0.05	-0.08
26	64	M	OS	1	22.74	PPV+PI	NA	on	on	off	on	on	20	23	0	22	21	26	28	27	26	26	+	+	+	+	+	0.10	-0.08
27	62	M	OD	1	26.52	SB	+	on	on	off	off	on	25	26	0	25	25	23	27	0	21	27	+	+	SRF	-	+	-0.08	0.05
28	57	M	OD	2	26.57	PPV	NA	on	off	on	on	on	27	0	21	23	25	30	27	27	25	31	+	+	+	+	+	-0.08	-0.08
29	65	M	OS	3	NA	PPV	NA	on	off	off	on	on	12	0	0	12	12	26	22	20	22	24	+	+	+	+	+	0.10	-0.08
30	74	M	OD	2	21.88	PPV+PI	NA	on	on	on	on	on	26	26	21	25	24	28	24	23	24	25	+	+	+	+	+	-0.08	-0.08
31	56	F	OS	2	23.52	PPV+PI	NA	on	on	off	on	on	29	28	10	26	27	29	23	29	25	29	+	+	+	+	+	-0.18	-0.08
32	56	M	OS	2	25.72	PPV+PI	NA	on	on	off	on	off	25	24	14	21	23	33	34	27	34	31	+	+	+	+	+	0.00	-0.08
33	56	M	OD	1	26.04	PPV+PI	NA	on	on	on	on	on	21	21	23	24	23	25	27	27	23	27	+	+	+	+	+	0.10	0.00
34	52	M	OD	1	26.25	PPV+PI	NA	on	on	on	on	on	22	19	22	21	22	30	30	28	28	30	+	+	+	+	+	0.05	-0.18

AL: axial length; Ez: ellipsoid zone; F: female; I: inferior; logMAR: logarithm of minimal angle of resolution; M: male; N: nasal; NA: not applicable; OD: oculus dexter; OS: oculus sinister
PI: phacoemulsification and aspiration + intraocular lens implantation; Post: postoperative; PPV: pars plana vitrectomy; Pre: preoperative; S: superior; SRF: subretinal fluid; T: temporal
